# A Consideration of Actinomycosis

**Published:** 1892-06

**Authors:** F. S. Billings

**Affiliations:** Director of the Patho-Biological Laboratoray of the State University of Nebraska


### A CONSIDERATION OF ACTINOMYCOSIS AS TO ITS NATURE AND RELATION TO THE PUBLIC HEALTH.

By Dr. F. S. Billings, Director of the Patho-Biological Laboratoray of the State University of Nebraska.

(*Concluded.*)

*Invasive Diseases Sometimes Contagious, but Never Infectious.*—The above statement, at first sight, appears to contradict a previous one, viz.: that while all contagious diseases are infectious, all infectious diseases are not, by any means, contagious. On the other hand, we must look for the evidence of the correctness of the statement in an entirely different direction ; one in which many invasive diseases resemble those of an infectious character, but in which infection itself does not occur, viz.: *Transmissibility*. It has been said already that from the clinical and experimental points of view that the word contagious is identical with transmissibility, that is, the specific cause can be transmitted from one animal to another, the nutrition in each case being favorable, and in the second produce disease, as in the first, the disease induced in the second animal, if of another species, need not necessarily present the same lesions as in the species in which the disease occurs as a natural affection. Invasive diseases are also of parasitic origin. These parasites are also obligatory or facultative in many cases. The obligatory invasive parasites which cause disease in animal life are all, I think, of an animal nature, while the facultative are limited to the vegetable kingdom. Those invasive diseases which are of an obligatory parasitic nature, are, in many cases, but by no means all, contagious, in the primary origin sense. For instance, the various forms of itch due to mites, are due to obligatory animal parasites, which only originate in or on, and frequently are limited to one species of animals, while others can be transmitted from the original species to others. These varieties of invasive diseases are transmissible, contagious, but here contagiousness, in the sense of many medical wonders of this century, is not always proven, in fact is often contradicted, by the inability to transmit to rabbits and guinea pigs, or other animals than those belonging to the original species. These great authorities would be justified, according to their reasoning in other cases, in asserting that the contagiousness was not proven, though contradicted by the daily experiences of the centuries. The itch mites of one species will seldom live in the skin of another ; the intestinal worms, tapes, have their peculiar hosts, outside the limits of which they will not live. Cysticercus cellulosa, the measle of the hog, is a small bladder embryo—if we take one from pork and transfer it to our mouths and swallow it, it becomes tænia solium : cysticercus bovis is a similar embryo in beef, and if we do the same with it we derive tænia mediocanellata. In both cases we have transmitted a living organism, which can produce disease from the hog or cattle to ourselves, and yet no man but a medical professor would have the affrontery to assert that a contagion had been transmitted, or that the produced disease was contagious because of the transmission. Trichiniasis can be transmitted from hogs to man in the same way, but is not contagious. Thus it is to be seen that in considering the real meaning of the words contagious or transmission, that they may have one significance in one place, and another in another, and none at all in other connections. In other words, we must first know what we are talking about. That something is *the natural primary origin of the cause of a given disease, original home of its parasitic cause, aud again, its natural course between individuals.*

These things have been all known for centuries, or must be definitely found out, before we begin to think of the investigation of such diseases in the laboratory, where we can only study the individual characteristics of the parasites, their peculiar biological phenomena and morphological variations, but to make the results of such studies practical we must be able by the study of the lesions produced in the animal body to theoretically trace the life of the action of the germs therein, and again, it exogenic germs, in the earth and animal surroundings. Laboratory investigations are but artificial aids by which to study the lives of disease-producing parasites in their natural habitat. Taken by themselves they may be strongly indicative, but must never be looked upon as conclusive evidence. The latter can only be obtained when through the former we arrive at a correct knowledge of the lives of such parasites in their natural habitats. The natural history of a disease, its origin and clinical course *give us its character. The laboratory* can establish its true cause and many points in the biology of the same.

*Report on Autopsy of State Farm Cow affected with Jaw Actinomycosis.*—Thoroughbred Hereford cow "Dianah," eleven years old, January 1, 1892 ; weight 1150 pounds. This cow has been more or less under my personal observation ever since I came to Nebraska, in 1886, and to my personal knowledge has produced four splendid calves, which are still healthy. A communication from the late foreman of the State Farm shows that this cow has been afflicted with jaw actinomycosis for over five years, and that no other case has occurred on the farm. This fact alone suffices to contradict any assertion that actinomycosis is a dangerous transmissible disease between diseased and healthy cattle. He says: "The Hereford cow Dianah (No. 2997, A. H. R.), according to the farm record was brought to the farm in 1882, and was there when I took charge in August, 1884, smooth and all right, but in the winter of 1884–5, she was hooked under the jaw by the cow standing next to her in the stable. Shortly after this I noticed a small lump growing on the jaw where she was hooked, and later on called a veterinarian to look at it. He called it "lump jaw," which it proved to be in very bad form, twisting the jaw out of shape. This is the first and only case of lump jaw in the farm herd. There has been from twenty to fifty herd of cattle in the herd from the time I took charge up to the present time, and the cow "Dianah" has been continually with the head in the pasture and in the feed lot, where we fed corn-fodder and hay to them. I have frequently seen matter discharging from the lump and drop on the ground and litters in the yard, and have seen this cow rubbing her jaw on the manger and edge of the water trough. We never took any precautions to keep her away from the other cattle while the lump was discharging. She has dropped six calves which are smooth and free from disease or any sign of lump jaw."

Her general physical condition has been as perfect as could be asked for, notwithstanding the nul-position of her jaws, due to atrophy of the masseter muscles on the afflicted side and consequent contraction on the other. She had become "cross-billed," as we say of fowls. The position of the jaws was such, so much distorted to one side, that the mouth did not close perfectly, and in the process of rumination more or less of her food would fall out on the ground and become mixed with the food or fodder of the other cattle. I have frequently seen the discharge from the lump, which always had severe fistulous opening in it, drop on the ground and on the corn-fodder which the cattle were eating, and when she went to drink she would rub the tumefaction on the edge of the trough and often press out matter not only on the edge, but it would run down on the water to which all the cattle had access in common. The enlargement itself was fully of the dimensions of a half-peck measure, though I am very sure it had grown smaller during the last year, a fact also noticed by Mr. Perrin, the present foreman of the farm. At the time we killed this cow, January 5, 1892, there was one fistulous opening still discharging a thick straw-yellow material. The scarcity of actinomycotic-clusters in this material as well as that filling pockets in the bone, was a matter of great astonishment to me, especially when taken into consideration with the enormous extent of the external lesions. This fact, however, in all probability accounts for the general diminishing of said lesions in extent during the past year, as mentioned above. The left jaw was the one complicated. The animal was knocked in the head and carefully dressed by a butcher in my presence and that of my assistant, Dr. F. W. Bremer, Dr. Giffin, one of the leading practitioners of Lincoln, and several other gentlemen.

*Necroscopy.*—On removing the skin all who were present were at once struck with the remarkable development of the sub-cutaneous fat tissue and the amount lining and covering those parts usually so lenseid, the abdominal cavity. Under the most exacting conditions the animal would have to be designated as a "fat" one. The cow was cut all to pieces and every muscle and every organ, and all the lymph glands most carefully examined, the same being afterwards subjected to a most exacting and detailed microscopic examination. Sections of these organs and muscles have been and are continually used in the laboratory for microscopic study, and yet not an iota of a lesion of any kind has been found in any of them. The muscles and organs were also subjected to most exacting microscopic examination immediately after slaughter, which extended over several hours, and before any post-mortal changes could occur, but in no instance could we find any pathological granulation, or any lesion of the protoplasm of the cells or fibres. *I can honestly and most emphatically affirm that if 100 or 1000 of the choicest short-horn beeves in the country were subjected to such an examination, every one of them having been in absolutely perfect health up to the time of slaughter, whether yearlings, two or three years old, or aged cattle, that not a single animal could possibly present a more perfect picture of absolute health in all its organs and muscles (except those of the immediately afflicted parts) than did this cow, even though she had had actinomycosis for over five years. Aside from the tissues of and around the complicated jaw, not even a microscopic speck of disease has yet been discovered in any organ or tissue, with the exception of two small urine-retention cysts about the size of a pea in one kidney. Not an indication of feverish meat could be seen anywhere.* My assistant,

Dr. Bremer, and friend, Dr. Griffin (both M. D.'s), each took one of the tenderloins home and consumed them in their own families.

That "fewer" lesions should not have been expected in this animal is evident by referring to the previously given table of temperature, taken for a period of two weeks prior to slaughtering.

Attention has been previously called to the astonishing scarcity of the clusters of actinomyces in the matter generally called pus, which exuded from a fistulous opening and was present in quite a number of small pockets in the jaw itself. That this material was not pus and that in general (in cattle at least) it is not pus, is shown in the result of the attempt to make cultures from the same. One hundred tubes representing glycerine-agor, glycerine-agor plus formate of soda; blood serum, and twenty-four glycerine-bouillon flasks were inoculated from this material, taken from a pocket in the jaw. *All but six remained absolutely sterile; those six were pusacultures of actinomyces.* These tubes were placed in the Thermostat at 37 C., about half in Buckner's Anacrobic mixture in hermetically sealed tubes. *Must assert that the actinomyces will develop at room temperature 70° F, on blood-serum, but not on other media.*

For us to-day *pus can no longer be considered any yellowish pus-like discharge. To be pus, such material contains pus-producing germs, and they must be demonstrated to be present in the material, both microscopically and by cultures.*

Cover-glass specimens of this material also gave negative results as to cocci or any suspicious forms of germ life, other than a few clusters of actinomyses, and some fragmentary developments of the same. The inoculation of animals in the ear and sub-cutaneously also gave negative results as to pus-producing germs. These results have a most important bearing upon that all important question, the exclusion or not of the flesh of such animals from consumption. The fact that no danger to consumers has ever resulted confirms the results in this examination, and that the actinomyses do not produce any irritable or dangerous chemical product, and hence have no deleterious effect on the general organism. My cultures corresponded with most of the illustrations given by Max Wolf and James Israel in Verchow's Archio, vol. 126, p 11, for the same cultivation-media (I did not use eggs), and as I am not engaged in the repetition of other people's work, and have no especial interest in bacteria-morphology when the points have been fairly well established, I refer those interested to the above and Bostroem's publication in Ziegler's "Beiträge Zür Pathologischen

Anatomie," vol. 9, 1891, as I shall publish nothing further on the morphology of this fungus and its biology, as that which has a pathogenic interest on plants is a matter for botanicali nvestigation, and not for a pathologist in animal tissues only. To return to our cow:

The skin covering the tumefaction presented quite a number of cicatricial contractions, marking the closed openings of former fistulæ; it was closely attached or united to the underlying tissue, which was thickened and indurated, being from one to two inches thick in many places. Another surprising fact was that in this enormous mass of pendulent tissue (as large as a half-peck measure) not a single pus center could be found, or one containing the previously noticed straw-colored material; the tumefaction consisted mostly of adipose tissue, separated into sections of various dimensions by dense septa of white connective tissue; the opening of the fistula out of which exuded the previously mentioned odorless straw-yellow material was in direct connection with a pipe or canal having dense, white, thick connective tissue walls, which directly connected with deeply-seated pocket in the jaw filled with the same material; several such cords could be traced extending through the mass of neoplastic tissue from the closed openings previously mentioned. The fat was bright yellow in color Immediately attached to the jaw and in the thickened connective tissue were numerous small centers of a delicate pinkish color, interrupted by large numbers of pinhead spots of a yellow color; sections of this tissue give most beautiful specimens of actively invading actinomyces are illustrated by Bostroum. On the external surface of the complicated jaw was a marked projection and callous, indicating that the jaw had been fractured at the time the cow was hooked, as indicated previously; the internal tissues of the jaw were spongy, with many centers of semi-gelatinous character; pockets filled with the non-odorous pus-like material and much more viscid than pus usually is, really having more of a thick mucuous character, were scattered here and there, some being quite small and none larger than a half-segment of dove's egg; the porotid gland on the afflicted side was indurated somewhat, but otherwise free from complications; all the lymph glands in the vicinity were entirely free of any actinomycotic complications; a few were atrophied or indurated from pressure, but the others were entirely normal. Tongue normal.

*Is the Flesh of Lump-jawed cattle to be condemed as unsuitable for human food?*—What is diseased meat? Were that question to be answered in the manner the language indicates, it would not be correct. Practically expressed, the question which should be asked is, *What must or may be considered disease-producing meat; that is, flesh which should be withheld from sale and condemned as unfit for human food?*

It may not be generally known to the public, and probably many physicians have not given any real thought to the subject, that all meat from diseased animals, even from animals having a notoriously dangerous contagious disease, is not considered as "diseased meat" when the animal is otherwise in good condition. For instance, thousands of pounds of meat from cattle diseased with contagious pleuro-pneumonia were allowed even by this very Illinois Live Stock Commission to be sold in Chicago when that disease prevailed there. Mind, I do not even insinuate any carlessness on the part of that commission in permitting its sale. They were right in doing so—when the general and special condition of the animal warranted it. In such cases, as in actino, a special examination of each carcass must be invariably made. In all European countries the same freedom of sale of the flesh of cattle having this disease (pleuro-pneumonia) is permitted, always dependent upon the nature of the lesions. On the other hand, if that flesh is subjected to either a microscopic or macroscopic examination, there is not a pathologist in the world who would say that the flesh was not "diseased flesh." If we turn to one of the highest and perhaps the most rigid authorities on this question of what meat is fit to eat, Schmidt Muelheim, Handbuch der Fleischkunde, p. 202, we shall find that the flesh from cattle having contagions pleuro-pneumonia is handled very briefly, only fifteen lines being given to it. He says: *The contagious pleuro-pneumonia of cattle is an exceedingly chronic infectious disease. The flesh of slaughtered animals has been consumed in many thousands of cases without any harm whatever resulting, even when the animal was in a condition of fully developed fever. The meat inspector must, however, carefully look out for gangrenous centers in the lungs, which often lead to septicæmia. In such cases the meat should be excluded from the market.*"

Baillet, another world-wide authority, in his Traité de l'inspection des Viandes de Boucherie, says: "*All practitioners admit, with common accord, that the usage of the flesh of animals attacked with contagious pleuro-pueumonia can be authorized without restriction. It has been established at the city of Lille alone that during a period of nineteen years the consumption of such flesh was not followed by a single accident; in that period 18,000 diseased cows were slaughtered and their flesh freely consumed.*

In this county thousands, one might be justified in saying millions, of hogs, diseased with swine plague, have been slaughtered and their flesh sold on the markets not only of this country, but the world, and yet not one single person is known to have become ill from it. Yet when we who know the nature of this disease (that it is a specific blood-poison septicæmia, and have seen the terrible lesions in the lungs and intestines), and see these things, the first and most natural sentiment is of the criminality of selling and slaughtering these hogs for human consumption. I must own that before this actinomycosis trial caused me to give special thought to the matter that no man more mercilessly condemed the criminality of this practice, though I had probably eaten much such pork and dispensed hundreds of pounds of it to sailors during my life as a seaman. Even though I had injected quite a dose of the bacilli of swine plaugue into my arm, and seen that no constitutional disturbances resulted, only a local swelling following, which soon subsided, still my continuous investigations of the disease kept me of the same opinion. Too much of the general condemnation of persons as well as other things is based upon really unreflecting sentiment, which is a most dangerous and unjust practice. We like that which we think we like, and only too often refuse to try things, even people which it would be very beneficial to us to have a far more intimate acquaintance with.

It has been clearly shown that meat diseased in itself, sa in the case of pleuro-pneumonia, swine plague, and even the majority of Texas cattle, is not necessarily disease producing when used as food by our own species. On the other hand, it can be clearly shown that flesh, which to the eye even of the expert butcher is absolutely healthy in appearance, can lodge an element which is most dangerous to us as food. Trichinous pork is alluded to. No condemnation, and so far as I know no examination either, is made in this country for the presence of the embryonal cysts of the armed and unarmed tape-worms in pork and beef respectively; the flesh is absolutely normal, and yet both these parasites are often dangerous and always troublesome customers to have in one's intestines. A man may almost die of trichinosis to-day and five years from now be perfectly healthy, so that the sharpest physical examination, and his own actions and true statement would fail to discover a sign of illness about him. He might poison himself, or be murdered, and yet the most exact optical consideration of his muscles—not microscopic—would fail in discovering an iota of evidence of disease; but if a cat or dog were given a piece of the same in the laboratory, most serious results to the animal would follow.

It is thus to be seen that this question as to what is diseased meat in reference to the public health is a most delicate one, and one not to be decided by ignorance, prejudicial sentiments or ignorant politicians.

With regard to actinomycosis in cattle, the striking facts were brought out at the late trial in Peoria, *that not one of the so-called "experts" for the Illinois Live Stock Commission had ever microscopically and scarcely macroscopically examined the flesh of any of the condemned animals, or of any of those parts of the body which under ordinary circumstances would naturally be sold in the market. I mean the muscular parts, back of the neck, including the anterior limbs, back, tenderloin and rump.*

In the only case of actino in the liver which I have seen, the sac was filled with a peculiar, viscid, yellow stuff that could not be called pus, *was a distension of a gall duct* and not a blood vessel, as any competent person could easily see, and the fungus must have been carried into the duct from the intestines by some worm parasite ; the connecting duct on each side of the "sac" was easily to be seen.

All cases of actino in the circulation thus far reported in men *have been on the venous side ;* this accounts for the immunity of the flesh at all places at all distant from the local lesion.

It is worthy ot note that several pathologists mention the presence of trichinae in the muscles of patients that have died of actino, *but not the actino ;* this shows pretty careful examination. There is not a reliable report of a single autopsy in cattle which gives an iota of evidence of the fungus getting into the arterial circulation, the only way it could get to the flesh of the trunk, and I do not know of a case where it has been reported that it can be safely said the thing was carried by the veins even.

Those experts who thought that they did a great thing in guessing at the difference in the diameters between the point of a club of actino and a red blood cell, forgot several things :

1. That in the club form the fungus is absolutely dead and harmless, and that it is the thread does the invading !

2. That the red blood cell has no fixity of dimensions in active circulation, but is an elastic body capable of adapting itself to the finest capillaries, whereas the end of the club is a hard, unelastic object that could not possibly pass where the red cells can

3. They forget that the club is not a round object, but a long one, and that its longitudinal diameter exceeds that of its point by a great many times; hence one end being heavier, it is very improbable that such an object could be carried very far in the circulation, it would get cross-ways at the first, branching off medium-sized vessels, not to speak of capillaries.

4. They did not know, experts though they imagine themselves, that to measure one point of one club would give no correct idea, and that these club points frequently represent a common root, like a man's arm, with three or more clubs, like fingers, at the end, so that they should have isolated one of these sections, and there they would have found a stiff, unchangable, sub-divided object as large as a dozen red blood cells.

Dr Hertwig, Director-General of the stock yards in the city of Berlin, wrote me:

1st. We do not condemn any cattle which are attacked with jaw antinomycosis. Only the diseased head is condemned.

2d. The case in which a whole beef would be condemned has only occurred once since inspection began here. In this case the head, tongue, liver, lungs, and lymph glands were all seriously invaded and the body in a very emaciated condition.

3d. So long as the trouble remains local, that is, confined to the head, and no general disturbance is present, we do not and shall not consider the meat of the balance of the body as unsuitable for consumption.

It should be known that these regulations are not the result of the personal observations and conclusions of Hertwig alone, but more especially of the Imperial Board of Health, which has the most competent corps of investigators in the world at its command and that they must also be sanctioned by the German Parliament.

One of the greatest feeders of cattle in Nebraska, as well as the world, Mr. Allen, manager of the Standard Cattle Company at Ames, where they feed five or six thousand head at a time, writes me "that he has never seen the disease extend among his cattle from those having the lump jaw, and that since the seizure and condemnation of such cattle in the market they slaughter all they have for home use, and have no compunctions about eating the meat, and that no ill effects have ever followed"

The greatest authorities in the world upon this question all coincide that the meat can be used with impunity if the diseased parts are thrown away and that there are no indications of disease in the marketable flesh itself.

Let us see what these authorities say:

There is a little volume entitled "Meat Inspection," written by Thomas Walley, M. R. C.V. S., Principal of the Edinburgh Royal (Dick's) Veterinary College, and Professor of Veterinary Medicine and Surgery. Before introducing the testimony of this witness a word as to his competency may be in order. In addition to a front rank among the veterinarians of Great Britain, an experience of thirty years in his profession and an official connection of over sixteen years with the Edinburgh abattoirs certainly qualify him to speak understandingly on this subject. This is his evidence ("Meat Inspection," pp. 128–129) :

The malady affects man, and is in him known as actinomycosis hominis, and while it has been transmitted by inoculation from man to the calf (Crookshank), so far as I am aware there is no direct evidence of the transmission of the disease from animals to man, nor indeed is such a contingency ever likely to arise, seeing that the vitality of the spores of the hyphomycetes is much inferior to those of other pathogenetic fungi, and that the changes produced by it in the organs attacked are so marked as to attract the immediate attention of even ordinary persons ; and what is of more importance, the lesions of the disease are seldom localized, except in the pig, in the muscular tissues of the body ; there may, however, be lesions in the muscles immediately contiguous to or connected with affected organs. The spores of the disease are supposed to gain access to the structure of the tongue and other organs by the medium of a break of surface, such as a wound, an abrasion, or a crack, and after so gaining access rapid development of the fungus goes on, while multiplication and dissemination of the spores take place. The irritation set up by the fungus leads to the production of a condition analogous to that seen in tuberculosis, viz., to a diffuse and chronic (interstitial) inflammation of the tissue of the organ, with the formation of nodules or granulations almost identical in morphological characters with those of tuberculosis. They are, however, larger than the miliary nodules of the latter disease, and do not, like them, tend to aggregate or coalesce, and consequently do not lead to the formation of such extensive lesions; and while calcification goes on in the centre of the nodule (constituting its core) caseation is much less pronounced. As has already been indicated, the glossal disease may invade the lips, the cheeks, and even the muscles and bones of the jaws and face, producing material changes in these parts, such as destruction of the cancellated portion of the bone, and opening up (rarefaction) of the compact tissue ; softening of the new growth goes on with the formation of imperfect abscesses, the contents of which may be evacuated through cloacæ in the bone, and finally by sinuses, through the soft tissues and the skin. The evacuation of this softened matter is followed by healing and the formation of a cicatrix ; hence the scars so frequently seen on the face and cheeks of affected animals. The internal organs may become invaded, primarily, through the medium of the various mucous membranes ; but the theory has been promulgated that a localized lesion may become, by virtue of the infectivity of the disease, systematically disseminated. Whether this is so or not is a matter of very little importance so far as our present purpose is concerned. In pigs, according to Johne, the actinomyces is frequently found in the tonsilar cavities, which may or may not have become the seat of any pathological change. Although certain organs, such as the tongue, may be largely involved, there is not much accompanying fever or interference with the normal functions or with the nutrition of the blood, and any systematic changes that arise are due mainly to annihilation of the functions of the tongue or of the particular organ involved, and as a result of this a state of poverty is induced. Seeing that the disease is rarely of a systemic nature, and that there is an absence of fever, the nutritive changes induced in the muscles are so slight as to do away, in the great majority of cases, with the question of nocuity of the flesh ; but notwithstanding this, if there is any evidence of malnutrition of the blood or of the flesh, the carcass should be condemned for human food, and in every instance the affected organ or organs should be effectually destroyed.

*Views Expressed at the International Congress of Hygiene at London, August, 1891.—Lancet*, Vol. 2, pp. 444-5. Actinomycosis was not really a new disease, but it was not recognized until comparatively recently as a specific micro-parasitic affection. There could be no doubt from the writings of Professor Dick that actinomycosis in cattle was extremely prevalent in Scotland from 1827 to 1839. In other parts of Great Britain it had probably been equally prevalent since, though lost sight of under various names, as wens, clyers, crewels, spina ventosa, carcinoma, &c. It was not until 1887, when Professor Crookshank established the etiology of wens, and investigated several outbreaks of a disease which proved to be actinomycosis, that it came to be generally recognized that actinomycosis in cattle was in some counties one of cattle in this country, and more recently the disease had been found to be equally prevalent in our Australian colonies and in the United States. Cases of antinomycosis in the human subject were met with at an early date. So long ago as 1848, M. Louis had a patient with pulmonary disease, supposed to be cancerous, and in pus from this case M. Lebert, of Zurich, discovered and accurately figured the tufts of club-shaped elements with which we are now so familiar. In late years nearly 200 cases had been described in the human subject, of which over 5 per cent. occurred in this couniry. Professor Crookshank thought there were two points especially worthy of discussion. In the first place, some manifestations of actinomycosis were still liable to be mistaken for tuberculosis. How far would this account for the very high percentage of cases of tuberculosis in cattle which had been reported by some observers? The question also arose, whether cases of actinomycosis ought to be placed in the same category as cases of tuberculosis, and condemned as unfit for food? If not, the distinction between the two diseases became a matter of practical importance, and the close similarity between the two diseases must be brought home to the minds of both medical and veterinary inspectors. With regard to inter-communicability of the disease between different species of animals, including man, the author had failed to find any evidence in support of the theory from clinical observation. He was not of the opinion that the disease was contagious in the ordinary sense of the term.

Professor Ponfick (Breslau) said that he agreed in the main with Professor Crookshank's conclusions. In cattle the disease was generally transmitted by means of the fodder, especially by straw. He thought that in both man and animals the source was the same, and that the disease did not spread directly from one to the other. Professor Nocard (Alfort) said that the observations of Professor Crookshank had been confirmed by all observers. He desired to dwell on the hygienic and sanitary side of the question. The geographical distribution of the disease was very irregular. Bavaria, Scotland, Italy, and some of the Northern States of America had been much affected, and at the Veterinary Hospital at Utrecht there were always cases of the disease. In France it was very rare, and generally remained in an isolated form. He had never seen cases transmitted by contagion or infection. The disease appeared to him undoubtedly to spread by means of certain food-stuffs. The wholesale destruction of the flesh of the affected beasts appeared to him to be quite unnecessary. It was towards the biology of the parasite that the hygienist's efforts should be directed, that some rational and efficient mode of prophylaxis might be arrived at.

M. Doyen (Rheims) read a paper on three cases of Actinomycosis in man, with lantern demonstration. During the last few months these observers have had occasion to examine in man three cases of actinomycosis, which in France, they say, is a very rare affection. If Liebert's case (1887) is excepted, the first observation was by Nocard and Leucet in 1888. Israel had, however, published three cases, and Ponfick one, and numerous other cases have been recorded in Germany before attention was called to the disease in France, in 1882. The authors had been on the outlook for the disease, but it was not until Feb., 1891, that they had the opportunity of observing their first two cases. The first patient consulted them for an indolent abscess of the jaw, with induration of the parotid. The cavity contained a bloody serous fluid in which were floating a dozen or more grains of actinomycosis; the whole mass was completely extirpated. The second case manifested itself as a deep phlegmonous induration at the base of the tongue, accompanied by a wooden-like hardness of the suprahyoid region. This case was cured by free incision, followed by curetting and antiseptic tampons.

As regards the etiology, these three cases all came from the environs of Rheims; they lived in the country, and one of them had acquired a habit of chewing grains of oats and of barley.

Dr. Serge Ivanov (Moscow, had been to many of the European abattoirs to study this affection, In discharge of his duties as chief inspector of the Moscow meat market, he had seen over 2,000 infected animals in the last two years. Most of these cases had been diagnosed in an early stage by the use of the microscope. Without this precaution, lesions in the shape of small tumors and abscesses might easily escape recognition. Dr. Goodall (Christchurch) had seen many cases of this affection, and he did not think there was sufficient evidence to justify the belief that the disease was communicable from animal to animal. It was worthy of note that it most commonly affected animals when they were about two years old and were changing their molar teeth. In one case he found a piece of heather firmly wedged between the teeth, and it was easy to see if such a structure were charged with the organism, how it might easily penetrate into the socket of the jaw beside the loosening tooth. Our present knowledge seemed to indicate that this was a fungus which found its normal habitat in vegetable structures, and was only accidental in animals. He felt sure that a careful investigation of the diseases communicable from the vegetable to the animal world would be productive of very fruitful results. Sir Henry Simpson remarked that in his district the disease was not endemic, and when imported, though it did not cease with the animals imported, yet it did not spread rapidly like a contagious disease. As to whether the meat from these carcases was fit for human consumption, he was content to permit its sale provided the locally diseased parts were cut away. Dr. George Fleming commented on the grave doubts which existed of the direct communicability of this disease to the human species. He thought that the legislature should not be overburdened in dealing with this matter, which could be controlled by ordinary measures. If any animal were in good condition and showed no fever, then its flesh was good to eat, but the actually diseased parts should not go into the market."

Here then, we have placed all the necessary evidence before our readers. A careful perusal of the same must have shown :

1st. That actinomycosis is not, genetically, a contagious disease, being facultative, parasitic, or exogenetic in origin, developing on some kind of vegetation.

2d. That it is not an infectious disease, because :

*a.* when uncomplicated it runs its course without fever.

*b.* it can be cured by a surgical operation every time that its location is favorably situated in man or beast.

3d. That transmission by inoculation has no necessary value in deciding the natural character or course of any disease as to its origin and relations in animals of the species in which it primarily occurs.

4th. That no lesions, either macroscopical or microscopical, have been found in any of the edible flesh at a point at any distance from the local center of invasion.

5th. That the fungus itself has never been found in flesh distant from the lesion in man, notwithstanding the most exact examination.

6th. That all persons who have any claim to be considered authorities agree in allowing that such flesh is good for human food, the diseased parts being removed and the animal being otherwise in equal condition to a corresponding one without lump-jaw affection.

7th. That the disease is an invasive, facultative, parasitic disease, not producing any chemical irritant or poison and hence incapable of producing any general constitutional disturbances.

8th. That diseased meat does not necessarily include the flesh of diseased animals, but rather such flesh as contains chemical, or other elements which of themselves are capable of producing disease in man when consumed as food.

In this regard the process of "ripening," as it is called, is on a parallel with disease, for as is well known, the "healthiest" meat when over-ripe is capable of causing the most serious disturbances in the human organism when eaten as food.

In this and the previous papers the full details and all the facts concerning actinomycosis in both man and beast have been placed before the reader, so far as they bear any relation whatever to the three interesting questions, viz.:

1. The contagiousness or not of the disease?
2. The infectiousness or not of the disease?
3. Whether the flesh is suitable for human food or not?

You can all form your conclusions, so thac it is scarcely necessary to say that there is not a particle of evidence which can be adduced, or which even can be so twisted as to confirm either the conclusion or actions of the Illinois Live Stock Commissioners.

It has been positively shown, that

1. The disease is not contagious in origin.
2. That it is not infectious in character.
3. That the flesh is perfectly safe for human food when the animal or carcass is in otherwise marketable condition.

---

TREVOSE, PENN.
APRIL 19, 1892.

MR. EDITOR:

It seems to me that Dr. Hickman has a hard time getting over Dr. William's criticisms. Now, suppose we look at this matter as a consumer (not from a scientific standpoint at all). I would like to know of what use is an inspector, if the inspector merely cuts off a diseased portion, then passes the remainder as sound; why, the butcher will do that without the inspector telling him. While, if the inspector passes such meat, then the butcher can look the purchaser in the face, and say, this is pure and sound, for the inspector said so. Now, I think the inspector's duty is to condemn all diseased cattle, no matter what the disease. Let the animals be brought into a healthy condition; if such cannot be done, then condemn to the vat.

W H. RIDGE.

